# MoS_2_/MWCNT Nanostructure: Enhanced Performance of Screen-Printed Carbon Electrode for Voltammetric Determination of 4-Nitrophenol in Water Samples

**DOI:** 10.3390/mi16040366

**Published:** 2025-03-23

**Authors:** Hadi Beitollahi, Somayeh Tajik

**Affiliations:** 1 Environment Department, Institute of Science and High Technology and Environmental Sciences, Graduate University of Advanced Technology, Kerman P.O. Box 76318-85356, Iran; 2 Research Center of Tropical and Infectious Diseases, Kerman University of Medical Sciences, Kerman P.O. Box 76169-13555, Iran; tajik_s1365@yahoo.com

**Keywords:** disposable sensor, carbon nanostructures, transition metal dichalcogenides, electrochemical sensor, 4-nitrophenol, water pollution

## Abstract

In the present work, we designed a straightforward and disposable voltammetric sensor utilizing a molybdenum disulfide/multi-walled carbon nanotube nanostructure-modified screen-printed carbon electrode (MoS_2_/MWCNTs/SPCE) for 4-nitrophenol (4-NP) determination. The successful synthesis of the MoS_2_/MWCNT nanostructure was characterized using Fourier transform infrared (FT-IR) spectroscopy, X-ray diffraction (XRD), transmission electron microscopy (TEM), and energy-dispersive X-ray spectroscopy (EXD) mapping. The electrochemical behavior of 4-NP at the MoS_2_/MWCNTs/SPCE was examined using differential pulse voltammetry (DPV), cyclic voltammetry (CV), and chronoamperometry techniques. The MoS_2_/MWCNTs/SPCE exhibited outstanding electro-catalytic activity for the voltammetric detection of 4-NP. Under optimized conditions, the reduction peak current showed a linear dependence with the concentration of 4-NP in the range of 0.05 to 800.0 µM, and a detection limit (LOD) of 0.01 µM was determined. In addition, the MoS_2_/MWCNTs/SPCE sensor has advantages including repeatability, reproducibility, stability, inexpensiveness, and practical application. The MoS_2_/MWCNTs/SPCE-based sensor was also utilized for the determination of 4-NP in real water specimens.

## 1. Introduction

Aromatic nitrophenol compounds hold significant importance and have been extensively utilized due to their role in the synthesis of a diverse array of products, including pharmaceuticals, pesticides, paints, synthetic dyes, and petrochemical products. Nitrophenols exhibit a range of properties, including toxicity, inhibitory effects, and bio-refractoriness [1,2,3]. Among these compounds, 4-nitrophenol (4-NP) is particularly significant due to its toxicity and hazardous nature, posing a threat to the health of living organisms in both aquatic environments and soil. The majority of fertilizer-manufacturing industries utilize 4-NP in the synthesis of insecticides for agricultural applications, and it also serves as an intermediate compound in the formulation of fertilizers. The plastic, dye, paper, and rubber industries significantly contribute to the prevalence of this pollutant in the environment. Consequently, it is inevitably discharged into the environment as industrial effluent, contributing to environmental degradation [4,5,6]. The initial symptoms of inhalation or ingestion 4-NP in humans include nausea, headache, cyanosis, and drowsiness. Additionally, it has been identified as a mutagen, potential carcinogen, and teratogen. Furthermore, the U.S. Environmental Protection Agency (EPA) lists 4-NP as a priority pollutant due to its toxic properties and persistence in the environment [7,8]. Consequently, the assessment of 4-NP is of significant importance for safeguarding the environment and mitigating any negative impacts on living organisms. Several methods are available for the determination of 4-NP, including spectrophotometry [9], fluorimetry [10], and chromatography [11] techniques. Nevertheless, these classical analytical approaches are often complex, costly, challenging to operate, and time-consuming. Based on the challenges of these methods, there is a need for using of methods that are cost-effective and straightforward in environmental analysis.

Electrochemical analysis techniques are increasingly favored over traditional techniques because they offer quick responses, ease of use, lower equipment costs, significant potential for miniaturization, and suitability for portable instrument applications. When optimizing electroanalytical methods for detecting specific species, it is essential to consider factors such as the solution nature, the polarographic or voltammetric technique employed, the electrode material, and the method of production. Electrochemical techniques typically rely on two processes the reduction or oxidation of the analyte [12,13,14,15,16,17]. Because the electroactive phenol and nitro groups in the 4-NP molecule exhibit electrochemical behavior during electrochemical processes, the currents can be utilized to detect low concentrations of nitrophenol compounds [18,19]. The surface properties of the electrode influence the performance of a created electrochemical sensor in detecting the target analyte. To achieve this, diverse working electrodes have been developed for the detection of different analytes, including carbon paste electrodes, bismuth film electrodes, glassy carbon electrodes (GCEs), and gold electrodes [20,21,22,23,24,25].

Screen printing technology is a firmly established methods that finds suitable application in the mass production of screen-printed electrodes (SPEs) for using in electrochemical sensors and biosensors. The significant advantages of this technology for construction of SPEs include its versatility, cost-effectiveness, ability for mass production, and the ability to construct customized electrode configurations with different substrates, shapes, geometries, and more. In the recent years, the screen-printed carbon electrodes (SPCEs) have found a suitable and efficient position in the electroanalysis of various compounds due to their advantages over conventional sensors, including their compact size, portability, ease of fabrication, and cost-effectiveness. SPCEs feature a very small working area, making them highly suitable for detecting trace amounts of target species. They are easy to operate, reliable, and offer high sensitivity [26,27,28,29,30]. The quick electrochemical process of various analytes on the bare electrodes surface necessitates a high over-voltage for their processes. To address these issues, incorporating an appropriate surface modifier to the electrode can be an effective solution. A suitable modifier can enhance the electron transfer between the electrodes and the electroactive species. Recently, there has been a focus on developing modified electrodes that exhibit excellent electro-catalytic properties and increased sensitivity for detecting target analytes [31,32,33,34,35,36].

Nanomaterials have been progressively developed and utilized because of their high specific surface area, favorable electro-catalytic properties, and outstanding conductivity. To date, different types of modified substances, including metal nanoparticles, carbon nanomaterials, ionic liquids (ILs), inorganic salt complexes, polymers, and nanocomposites have been reported. These materials were synthesized either physically or chemically and were used to fabricate modified electrodes for analytical determinations [37,38,39,40,41]. Molybdenum disulfide (MoS_2_) is a compound that composed of Mo and S atoms arranged in a layered structure. MoS_2_ is a type of transition metal dichalcogenides (TMDs) that shows interesting features, including semiconducting behavior, optical properties, mechanical strength, and other properties. Due to these features, it has an efficient potential for various applications. MoS_2_ has attracted attention in various fields, including electrochemical devices, catalysis, hydrogen storage, capacitors and sensors [42,43,44]. However, it is important to consider its application as a sensor electrode material, as MoS_2_ still exhibits lower electronic conductivity compared to MWCNTs and graphene. Combining MoS_2_ with other conductive materials may help to mitigate this limitation [45,46].

The materials based on carbon have been widely used for a range of applications, including super-capacitors, adsorbents, batteries, catalysts, and electrodes. To improve electrochemical performance, multi-walled carbon nanotubes (MWCNTs) were incorporated. Because of their high specific surface area and excellent conductivity, MWCNTs have been extensively used as a modifier. In fact, MWCNTs provide outstanding electrical conductivity and physicochemical properties, which enhance electron transport between target analytes and surface of the electrodes [47,48,49,50].

In this study, a MoS_2_/MWCNTs nanostructure was designed and fabricated using a simple method. The morphologies and crystal structures of the synthesized MoS_2_/MWCNTs nanostructure were analyzed using various testing techniques. Then, the MoS_2_/MWCNT nanostructure-modified SPCE was applied for electroanalysis of 4-NP. The electro-catalytic and electrochemical properties of the MoS_2_/MWCNTs/SPCE were investigated using CV, chronoamperometry, and DPV techniques. Moreover, the electro-catalytic properties of MoS_2_/MWCNTs enhanced the sensitivity of the sensor for 4-NP. Besides, low LOD, wide linear range, good repeatability, reproducibility, good selectivity, and long-term stability were acquired. Finally, the detection of 4-NP in practical samples was conducted using water samples on the MoS_2_/MWCNTs/SPCE under optimal conditions.

## 2. Experimental Section

### 2.1. Reagents and Instruments

All reagents were obtained in their highest general purity and employed as received, without additional purification. The phosphate-buffered solutions (PBSs) were prepared using ortho-phosphoric acid (0.1 M) and pH adjustment of the solutions within the range of pH 2.0 to pH 9.0 was performed using NaOH solution. They were employed as the supporting electrolyte.

CV, chronoamperometry, and DPV experiments were conducted using the PGSTAT302N electrochemical workstation (Metrohm, Herisau, Switzerland). Electrochemical investigations were conducted using disposable screen-printed carbon electrodes (SPCEs) from Dropsens, which include carbon working electrode (DRP-110) along with a carbon counter electrode and a silver-pseudo reference electrode. Solutions were prepared using deionized water from Millipore, Direct-Q 8UV (Darmstadt, Germany), and were freshly made each day.

The morphological investigations were carried out by using an EM 208S transmission electron microscope (TEM, Philips, The Netherlands). The XRD patterns were obtained using an X-ray powder diffractometer (Panalytical, X’Pert Pro, Etten Leur, The Netherlands).

### 2.2. Synthesis of MoS_2_/MWCNT Nanostructure

The MoS_2_/MWCNT nanostructure was prepared through the solvo/hydrothermal treatment of relevant precursors. Initially, 70 mg of functionalized MWCNTs (MWCNTs-COOH) was dispersed into 35 mL of deionized water and 35 mL of DMF and ultrasonicated for about 1.5 h to form a well-dispersed suspension. Then, 0.240 mmol Na_2_MoO_4_·2H_2_O (0.0580 g) and 0.912 mmol thiourea (0.0694 g) were poured into the suspension of MWCNTs under stirring conditions. The stirring process was continued for 50 min at ambient temperature. Subsequently, the resulting suspension was poured into the solvo/hydrothermal autoclave reactor, which was heated into an oven at a temperature of 200 °C for 24 h. The autoclave was naturally cooled to ambient temperature after the solvo/hydrothermal reaction was completed over the certain time. After that, the centrifugation step at 6000 rpm for 10 min was performed to collect the black precipitates. Also, in order to wash the precipitates with deionized water and ethanol, the centrifugation step was repeatedly performed several times. Finally, the vacuum-drying process of the as-prepared MoS_2_/MWCNT nanostructure was carried out for 15 h in an oven by maintaining a constant temperature of 65 °C.

### 2.3. Modification Process of SPCE

For the modification of SPCE, an aqueous suspension was prepared through dispersing 1 mg of MoS_2_/MWCNT nanostructure in 1.0 mL of deionized water. The suspension was then ultra-sonicated for 30 min to achieve uniform dispersion of the nanostructure powder. The SPCE was modified by applying 4.0 μL of the resulting suspension and allowing them to dry at ambient temperature.

### 2.4. Preparation of Real Water Specimens

Two distinct water specimens, namely tap water and river water, were chosen as real specimens. The specimen’s solutions were filtered using a 0.45 μm membrane filter. When 4-NP was not spiked into the water specimens, the electrochemical response was not detected. This case indicated that 4-NP was not present in the water specimens. Therefore, 4-NP was added to the water specimens to evaluate the electrochemical performance of the MoS_2_/MWCNTs/SPCE sensor. Firstly, 5 mL of the water specimens was collected and diluted with 15 mL of phosphate-buffered solution (0.1 M, pH 7.0) in the voltammetric cell. Subsequently, 4-NP was added at certain concentrations. Finally, utilizing the standard addition technique, the 4-NP concentration was determined.

## 3. Results and Discussion

### 3.1. Structural and Morphological Characterization of MoS_2_/MWCNTs Nanostructure

**Figure 1:**
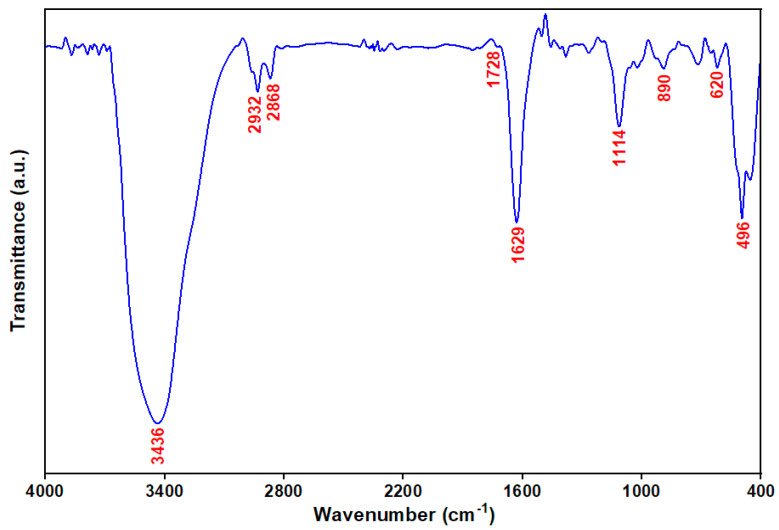
FT-IR spectrum of MoS_2_/MWCNT nanostructure.

The FT-IR spectrum of MoS_2_/MWCNT nanostructure is shown in Figure 1. The FT-IR spectrum demonstrates the existence of C=O, C-O, and OH functional groups at 1728 cm^−^^1^, 1114 cm^−^^1^, and 3436 cm^−^^1^ from -COOH groups in the structure of MWCNTs [51,52,53]. The stretching vibration of C=C groups also appeared at 1629 cm^−^^1^, which indicates the graphite structure of MWCNTs. The absorption bands at 2932 cm^−^^1^ and 2868 cm^−^^1^ could be related to the characteristic symmetric and asymmetric stretching vibrations of CH_2_ groups produced at the defect sites on the surface of acid-oxidized MWCNTs [54,55]. Moreover, the observed absorption bands at 496 cm^−^^1^, 620 cm^−^^1^, and 890 cm^−^^1^ are assigned to the stretching vibrations in MoS_2_. Therefore, these absorption bands reveal the formation of a MoS_2_/MWCNT nanostructure.

**Figure 2:**
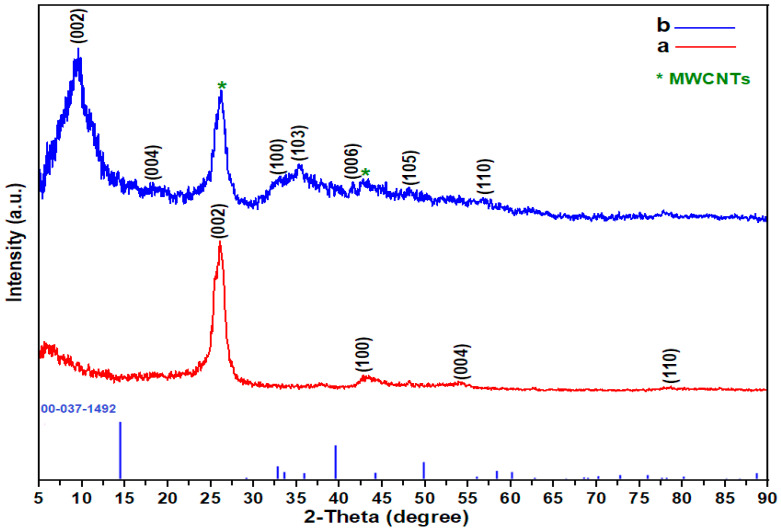
XRD patterns of MWCNTs-COOH (a) and MoS_2_/MWCNTs nanostructure (b).

The XRD characterization of the MWCNTs-COOH and MoS_2_/MWCNT nanostructure was carried out and the corresponding XRD patterns are presented in Figure 2. The XRD pattern of MWCNTs-COOH (Figure 2a) demonstrates a sharp peak at 2θ of 26.0° and other peaks at 43.1°, 53.8°, and 78.3°, which correspond to the (002), (100), (004), and (110) planes of the graphite structure, respectively. The XRD pattern of the MoS_2_/MWCNT nanostructure is exhibited in Figure 2b. The diffraction peaks for MoS_2_ are located at 2θ = 32.8°, 35.3°, 41.6°, 48.3°, and 57.3°, corresponding to the (100), (103), (006), (105), and (110) planes of MoS_2_, respectively. However, the characteristic peak for the (002s) plane of hexagonal MoS_2_ (2H phase (JCPDS No. 00-037-1492)) was not detected, while the diffraction peak of this plane shifted to a lower 2θ at 9.2°, demonstrating a significant increase in interlayer distance. Also, the peak located at 2θ = 18.2° (004) is further evidence of increased interlayer distance. These observations are similar to the results reported in previous works [56,57,58,59]. Furthermore, the diffraction peaks of MWCNTs can be seen in the XRD pattern of the MoS_2_/MWCNT nanostructure.

**Figure 3:**
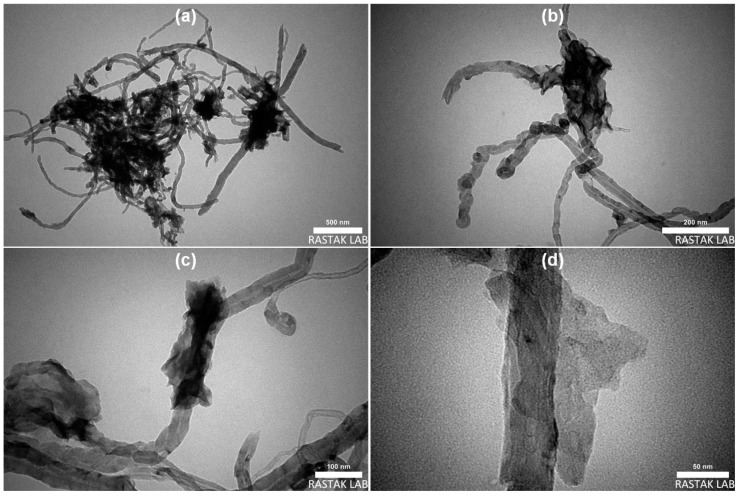
TEM images of MoS_2_/MWCNT nanostructure at various magnifications (scale bar: 500 nm (**a**), scale bar: 200 nm (**b**), scale bar: 100 nm (**c**), and scale bar: 50 nm (**d**)).

The morphological features of the as-prepared sample were studied by obtaining TEM images. The TEM images are presented in Figure 3. From the TEM images, we see that MoS_2_ possesses a sheet-like structure with a thin thickness. Also, it can be observed that MoS_2_ NSs grew around the MWCNTs. From these images, it can be deduced that both MoS_2_ NSs and MWCNTs are present in the prepared sample.

**Figure 4:**
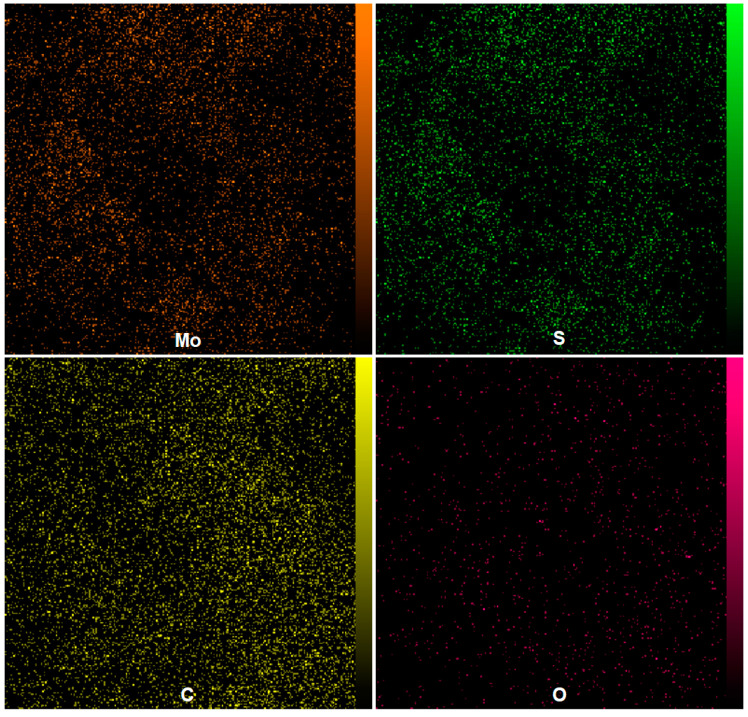
EDX mapping images of Mo, S, C, and O elements in the MoS_2_/MWCNT nanostructure.

The elemental compositions of the as-prepared MoS_2_/MWCNTs nanostructure were investigated using EDX mapping images (Figure 4). The elemental mapping images indicate the presence and distribution of Mo, S, C, and O elements in the prepared nanostructure, in illustrated in Figure 4. Also, no other impurity was detected in the nanostructure.

### 3.2. Electrochemical Behavior of 4-NP at the MoS_2_/MWCNTs/SPCE

**Scheme 1:**
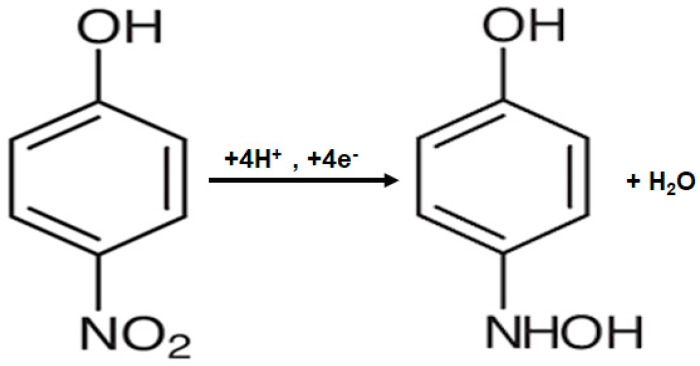
The suggested mechanism for the determination of 4-NP at the MoS_2_/MWCNTs/SPCE.

The effect of pH on the electro-catalytic activity of 4-NP at the MoS_2_/MWCNTs/SPCE was investigated in diverse pH solutions ranging from 2.0 to 9.0, each containing 50.0 µM 4-NP. It was observed that the reduction signals of 4-NP increased as the pH values rose from 2.0 to 7.0, followed by a slight decrease with further increases in pH. The highest cathodic peak current was achieved at pH = 7.0, which was subsequently used in this study. Scheme 1 depicts the suggested mechanism for the determination of 4-NP at the MoS_2_/MWCNTs/SPCE.

**Figure 5:**
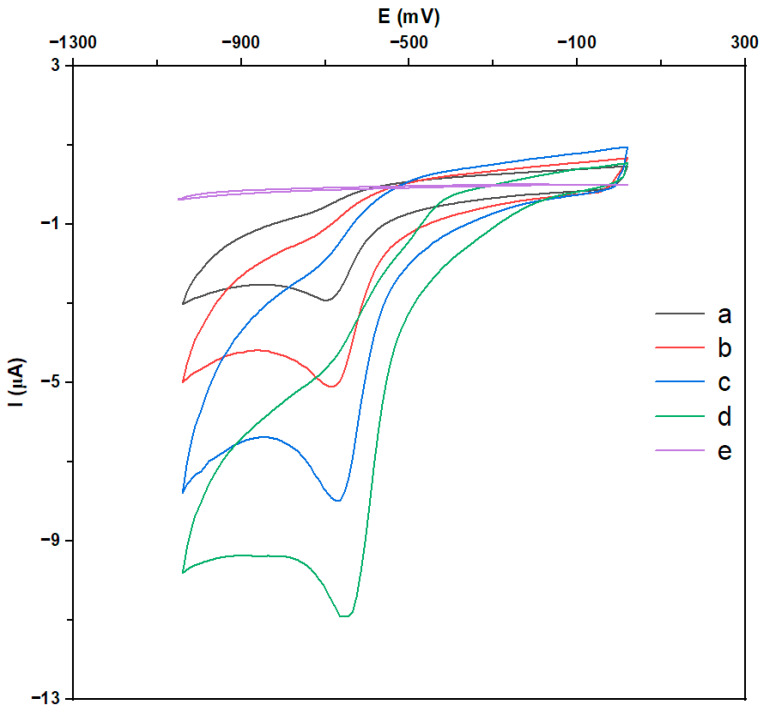
CVs of bare SPCE (a), MoS_2_/SPCE (b), MWCNTs-COOH/SPCE (c), and MoS_2_/MWCNTs/SPCE (d) in PBS: 0.1 M, pH 7.0 (containing 200.0 µM 4-NP), and MoS_2_/MWCNTs/SPCE in PBS: 0.1 M, pH 7.0 without 4-NP (e) (scan rate: 50 mV/s).

Figure 5 displays the comparative CV responses of the bare SPCE, MoS_2_/SPCE, MWCNTs-COOH/SPCE, and MoS_2_/MWCNTs/SPCE recorded in PBS (0.1 M with a pH of 7.0) containing 200.0 µM 4-NP, at a fixed scan rate of 50 mV/s. From CV studies, we can observe that bare SPCE exhibits a poor response to 4-NP with a low reduction peak current (Ipc = −2.9 µA) (cyclic voltammogram a). From cyclic voltammogram b, the reduction peak of 4-NP exhibits a higher peak current (Ipc = −5.1 µA) at the surface of the MoS_2_-modified SPCE in comparison with the bare SPCE, indicating the beneficial effect of MoS_2_ NSs in improving the efficiency of bare SPCE. However, on the surface of the MWCNTs-COOH-modified SPCE (cyclic voltammogram c), higher Ipc (−8 µA) was recorded compared to the MoS_2_/SPCE, which was attributed to the high electrical conductivity of MWCNTs. Also, the MoS_2_/MWCNTs/SPCE exhibited a significant current response (Ipc = −10.9 µA) at relatively lower over-potential (reduction peak potential (Epc) = −640 mV) for 4-NP compared to the bare SPCE, MoS_2_-modified SPCE, and MWCNTs-COOH-modified SPCE, as demonstrated in cyclic voltammogram d. It was shown that the combination of MoS_2_ NSs and MWCNTs played a more effective role in improving the efficiency of the modified electrode. Overall, the improved Ipc and decreased Epc contributed to the synergetic effect of two components of MWCNTs and MoS_2_ NSs, leading to efficient electron transfer capability and improved catalytic performance. Furthermore, it can be clearly observed that there is no reduction peak in PBS 0.1 M without 4-NP on the MoS_2_/MWCNTs/SPCE (cyclic voltammogram e).

### 3.3. Influence of Scan Rate

**Figure 6:**
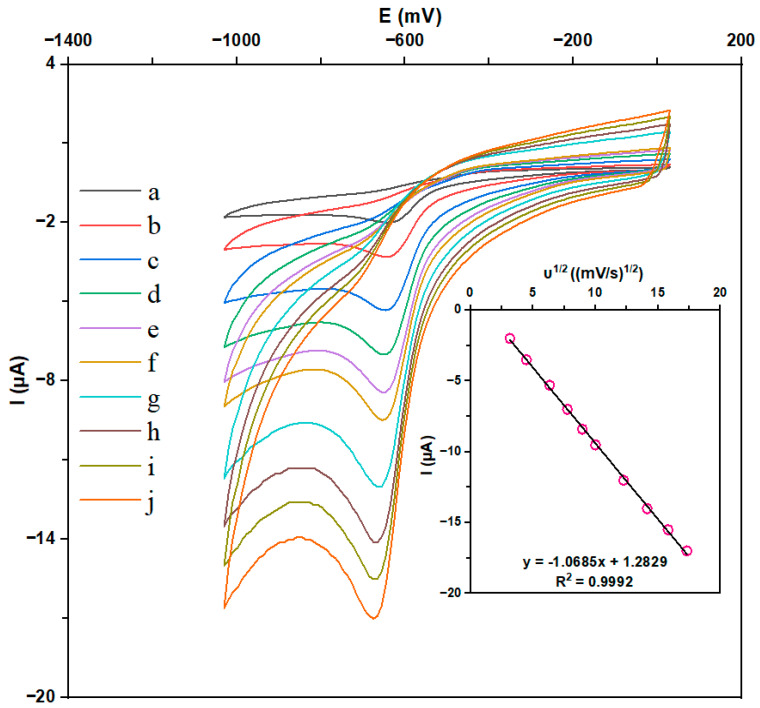
CVs of MoS_2_/MWCNTs/SPCE in 0.1 M PBS (pH: 7.0) containing 100.0 µM 4-NP at diverse scan rates from 10 to 300 mV/s (CVs of a to j correspond to (a) υ 10 mVs^−1^, (b) υ 20 mVs^−1^, (c) υ 40 mVs^−1^, (d) υ 60 mVs^−1^, (e) υ 80 mVs^−1^, (f) υ 100 mVs^−1^, (g) υ 150 mVs^−1^, (h) υ 200 mVs^−1^, (i) υ 250 mVs^−1^, and (j) υ 300 mVs^−1^). Inset: plot of Ipc vs. υ^1/2^.

Figure 6 exhibits the influence of the potential scan rate on the CV responses of the MoS_2_/MWCNTs/SPCE towards the reduction process of 4-NP (100.0 µM). The cyclic voltammograms at various scan rates demonstrate that increasing the scan rate causes an increase in the Ipc. At the same time, by increasing the scan rate, the cathodic peak potential (Epc) shifts slightly towards more negative values. Based on a detailed analysis of the cyclic voltammograms, a linear plot of Ipc as a function of the square root of the scan rate (υ^1/2^) is observed (regression equation: Ipc (μA) = −1.0685 υ^1/2^ (mV s^−1^)^1/2^ + 1.2829 (R^2^ = 0.9992)) (Figure 6 (Inset)). This linear relationship demonstrates a typical diffusion-controlled electrochemical process of 4-NP.

### 3.4. Chronoamperometric Studies

**Figure 7:**
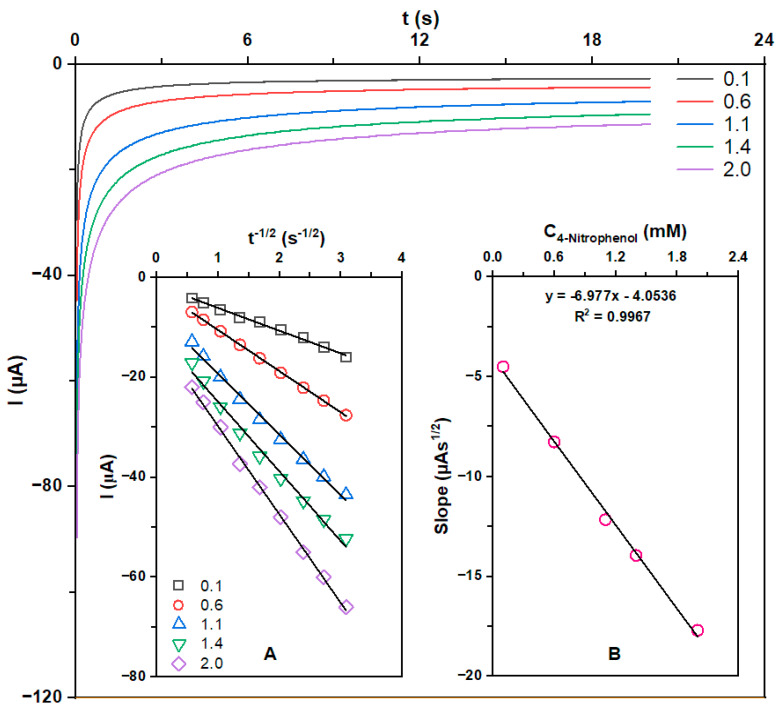
Chronoamperograms recorded at the MoS_2_/MWCNTs/SPCE in 0.1 M PBS (pH = 7.0) for various concentrations of 4-NP from 0.1 mM to 2.0 mM (potential step at −700 mV). Inset (**A**): Plots of I vs. t^−1/2^ based on the observed chronoamperograms. Inset (**B**): A linear plot of the slopes for the straight lines in Inset A vs. the concentration of 4-NP.

Given that the reduction peak of 4-NP is a diffusion-controlled process, we employed the chronoamperometry approach to assess the diffusion coefficient (D) of 4-NP at the MoS_2_/MWCNTs/SPCE. Figure 7 displays chronoamperograms of various solutions containing concentrations of 4-NP, ranging from 0.1 to 2.0 mM, in PBS (pH = 7.0) at the MoS_2_/MWCNTs/SPCE surface (chronoamperograms were recorded at potential step = −700 mV). For an electro-active analyte (such as 4-NP) with a diffusion coefficient of D, the electrochemical reaction current is described by the Cottrell equation:

I = nFACD^1/2^П^−1/2^t^−1/2^ (I: current (µA), n: number of electrons participating in the electrochemical process of the target compound, F: Faraday’s constant (96485 C/mol), A: the surface area of the working electrode (cm^2^), C: the concentration of the target compound (mol/cm^3^), D: diffusion coefficient (cm^2^/s), and t: time (s)).

According to the Cottrell equation (under diffusion-limited transport), a plot of I vs. t^−1/2^ is linear. We plotted the variation in I as a function of t^−1/2^ for different chronoamperograms (Figure 7A). Then, we plotted the slopes of Cottrell plots in Inset A vs. concentrations of 4-NP (Figure 7B). Therefore, based on the Cottrell equation and using the obtained slope in Inset B, the diffusion coefficient of 4-NP was found to be 1.04 × 10^−6^ cm^2^/s.

### 3.5. Analytical Performance of MoS_2_/MWCNTs/SPCE

**Figure 8:**
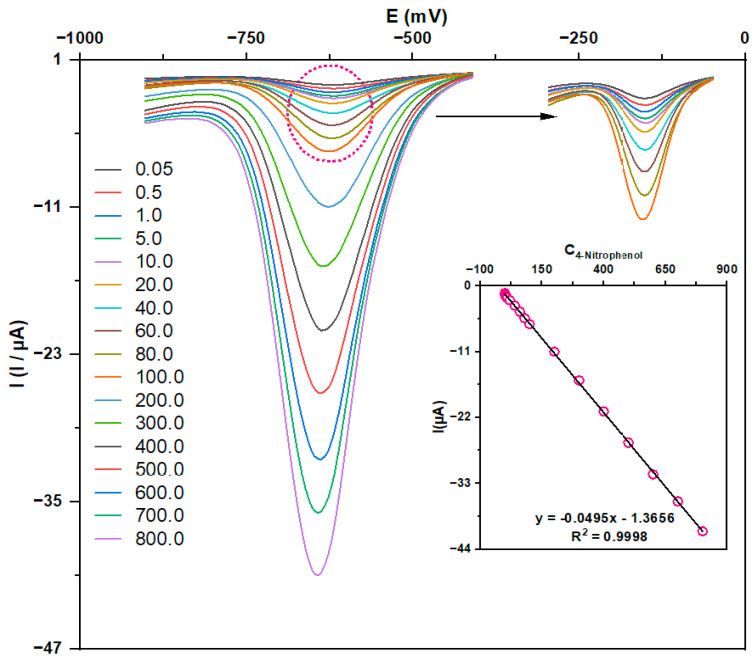
DPVs of MoS_2_/MWCNTs/SPCE in diverse concentrations of 4-NP ranging from 0.05 to 800.0 µM in 0.1M PBS at pH = 7.0. Inset: Plot of Ipc vs. concentration for 4-NP determination.

The DPV method was employed to quantify 4-NP in 0.1 M PBS at the MoS_2_/MWCNTs/SPCE. Figure 8 shows the DPVs of 4-NP obtained through DPV for different concentrations ranging from 0.05 to 800.0 µM. The reduction peak currents of 4-NP increased with rising concentrations. The reduction peak current of 4-NP is plotted versus its concentration in the inset of Figure 8. It demonstrates linearity from 0.05 to 800.0 µM. This relationship can be represented with the following regression equation: Ipc (ìA) = −0.0495C_4-NP_ − 1.3656 (R^2^ = 0.9998). The limit of detection (LOD) was calculated based on the equation LOD = 3S_b_/m, where S_b_ is the standard deviation of measurements of the blank sample, and m is the slope of the calibration curve. Based on the above equation, it was found that the value of LOD was 0.01 µM. Also, the obtained limit of quantification (LOQ) (LOQ = 10SD/m) was 0.033 µM.

Also, an investigation for comparing the sensing ability of the MoS_2_/MWCNTs/SPCE with some modified electrodes in the literature was carried out, and the important analytical parameters (linear range and LOD) of these works are presented in Table 1. From the summarized results in Table 1, the MoS_2_/MWCNTs/SPCE presented here demonstrates a lower LOD and wider linear range for 4-NP levels compared to previous studies. Therefore, from Table 1, it can be deduced that the MoS_2_/MWCNT nanostructure serves as an efficient platform for determining 4-NP, and the enhanced ability of the fabricated sensor could be related to the electro-catalytic activity of MoS_2_/MWCNT nanostructures.

### 3.6. The Repeatability and Reproducibility of the MoS_2_/MWCNTs/SPCE Sensor

The fabricated electrode (MoS_2_/MWCNTs/SPCE) demonstrated significant repeatability, exhibiting a relative standard deviation (RSD) of 4.1% across seven successive measurements conducted with a single electrode. Furthermore, the sensor showed satisfactory reproducibility, with an RSD of 3.9% across seven independent measurements performed on seven distinct electrodes. The findings indicate that the MoS_2_/MWCNTs/SPCE sensor has satisfactory levels of repeatability and reproducibility in measuring 4-NP.

### 3.7. Response Stability

The stability of the developed sensor (MoS_2_/MWCNTs/SPCE) was studied by keeping the MoS_2_/MWCNTs/SPCE sensor at room temperature for 3 weeks and detecting 4-NP. After this timeframe, the sensor demonstrated a retention rate of 96.7% of its initial response, thereby suggesting a good level of storage stability for the MoS_2_/MWCNTs/SPCE.

### 3.8. Selectivity of the MoS_2_/MWCNTs/SPCE Sensor

Selectivity demonstrates the performance of a designed sensor in terms of the response to a specific compound in the presence of other compounds. Therefore, we investigated the sensor’s selectivity in response to 4-NP. DPV was conducted using the MoS_2_/MWCNTs/SPCE to detect 50.0 µM of 4-NP in the presence of various potential interferents in 0.1 M PBS (pH = 7.0). The results demonstrated that equal concentrations of hydroquinone, catechol, 4-aminophenol, and resorcinol did not significantly impact the detection of 4-NP, as the signal changes were below 5%. Also, the equal concentrations of K^+^, Na^+^, Mg^2+^, Ca^2+^, Cu^2+^, Cl^−^, Br^−^, SO_4_^2−^, and NO_3_^−^ did not significantly impact the detection of 4-NP, as the signal change remained below 5%. Table 2 exhibits the interference effect of the investigated compounds on the change in the cathodic peak current of 4-NP (50.0 µM).

### 3.9. Practical Application of the MoS_2_/MWCNTs/SPCE Sensor for 4-NP Detection

To illustrate the suitability and potential of the MoS_2_/MWCNTs/SPCE for practical specimen analysis, the suggested method was employed to quantify 4-NP in real tap water and river water specimens. The investigation of 4-NP content in the real specimens was conducted using the DPV technique along with the standard addition approach. The results for the real specimens are presented in Table 3. The recoveries of the spiked specimens ranged from 96.0% to 104.3%, indicating that the MoS_2_/MWCNTs/SPCE is practical and could be applied for 4-NP determination in real water specimens.

## 4. Conclusions

In this work, we reported the design of a MoS_2_/MWCNT nanostructure-modified SPCE and its utilization for the voltammetric determination of 4-NP. The developed sensor (MoS_2_/MWCNTs/SPCE) demonstrated strong electrical performance and delivered an amplified response for the determination of 4-NP. Additionally, the voltammetric determination of 4-NP was assessed using the MoS_2_/MWCNTs/SPCE, with a linear concentration range from 0.05 to 800.0 µM and an LOD of 0.01 µM. The proposed disposable sensor displayed an excellent sensitivity of -0.0495 µA/µM. The MoS_2_/MWCNTs/SPCE sensor has good stability, reproducibility, repeatability, and selectivity for the determination of 4-NP. Furthermore, the MoS_2_/MWCNTs/SPCE has the potential to be utilized for 4-NP determination in real water specimens, positioning the developed electrode as a promising option for practical applications.

## Figures and Tables

**Table 1:** Analytical parameters of the developed sensor (MoS_2_/MWCNTs/SPCE) in comparison to other works for 4-NP detection.

Electrochemical Sensing Platform	Electrochemical Technique	Linear Range	LOD	Ref.
Chitosan/ZnO nanoneedle-modified SPCE	DPV	0.5 to 400.6 µM	0.23 µM	[8]
Activated carbon-modified glassy carbon electrode (GCE)	Linear sweep voltammetry	1.0 to 500.0 µM	0.16 µM	[60]
Chitosan-modified carbon paste electrode (CPE)	DPV	0.1 µM to 0.1 mM	0.093 µM	[61]
3, 5- diamino-1,2,4- triazole film-modified gold electrode	DPV	0.24 to 130.6 µM	0.09 µM	[62]
Nb2CTX/Zn, Co-embedded N-doped carbon nanocage-modified GCE	DPV	1.0 to 500.0 µM	0.07 µM	[63]
Cu0.5–Fe3O4 @ carbon black Vulcan-72-modified GCE	DPV	0.1 to 4.0 µM and 5.0 to 150.0 µM	0.065 µM	[64]
Pyridine diketo pyrrolopyrrole-functionalized graphene oxide-modified GCE	DPV	0.5 to 163.0 µM	0.1 µM	[65]
MoS_2_/MWCNTs/SPCE	DPV	0.05 to 800.0 µM	0.01 µM	Present work

**Table 2:** Interferences of different compounds on the cathodic peak current of 4-NP (50.0 µM).

Interfering Compounds	Change in Cathodic Peak Current (%)
Hydroquinone	+3.5
Catechol	+3.8
4-aminophenol	+4.3
resorcinol	+2.9
K^+^	−1.6
Na^+^	−1.9
Mg^2+^	−0.7
Ca^2+^	−0.8
Cu^2+^	−2.3
Cl^−^	+0.6
Br^−^	+1.1
SO_4_^2−^	−1.3
NO_3_^−^	+2.2

**Table 3:** Determination of 4-NP at MoS_2_/MWCNTs/SPCE in real samples (n = 5).

Sample	Concentrations of 4-NP		Results	
	Spiked (µM)	Found (µM)	Recovery (%)	RSD (%)
Tap water	0	-	-	-
	5.0	4.9	98.0	3.3
	7.0	7.3	104.3	1.9
	9.0	9.1	101.1	2.7
	11.0	10.7	97.3	2.3
River water	0	-	-	-
	5.5	5.6	101.8	2.2
	7.5	7.2	96.0	3.0
	9.5	9.7	102.1	2.4
	11.5	11.4	99.1	2.8

## Data Availability

The data presented in this study are available on request from the corresponding authors.
